# Intrinsic Disorder in Plant Transcription Factor Systems: Functional Implications

**DOI:** 10.3390/ijms21249755

**Published:** 2020-12-21

**Authors:** Edoardo Salladini, Maria L. M. Jørgensen, Frederik F. Theisen, Karen Skriver

**Affiliations:** REPIN and the Linderstrøm-Lang Centre for Protein Science, Department of Biology, University of Copenhagen, DK-2200 Copenhagen, Denmark; edoardo.salladini@bio.ku.dk (E.S.); mjoergensen@bio.ku.dk (M.L.M.J.); frederik.theisen@bio.ku.dk (F.F.T.)

**Keywords:** ID, activation domain, interactome, coupled folding and binding, phase separation, posttranslational modification, electrostatic interactions, sequence motif

## Abstract

Eukaryotic cells are complex biological systems that depend on highly connected molecular interaction networks with intrinsically disordered proteins as essential components. Through specific examples, we relate the conformational ensemble nature of intrinsic disorder (ID) in transcription factors to functions in plants. Transcription factors contain large regulatory ID-regions with numerous orphan sequence motifs, representing potential important interaction sites. ID-regions may affect DNA-binding through electrostatic interactions or allosterically as for the bZIP transcription factors, in which the DNA-binding domains also populate ensembles of dynamic transient structures. The flexibility of ID is well-suited for interaction networks requiring efficient molecular adjustments. For example, Radical Induced Cell Death1 depends on ID in transcription factors for its numerous, structurally heterogeneous interactions, and the JAZ:MYC:MED15 regulatory unit depends on protein dynamics, including binding-associated unfolding, for regulation of jasmonate-signaling. Flexibility makes ID-regions excellent targets of posttranslational modifications. For example, the extent of phosphorylation of the NAC transcription factor SOG1 regulates target gene expression and the DNA-damage response, and phosphorylation of the AP2/ERF transcription factor DREB2A acts as a switch enabling heat-regulated degradation. ID-related phase separation is emerging as being important to transcriptional regulation with condensates functioning in storage and inactivation of transcription factors. The applicative potential of ID-regions is apparent, as removal of an ID-region of the AP2/ERF transcription factor WRI1 affects its stability and consequently oil biosynthesis. The highlighted examples show that ID plays essential functional roles in plant biology and has a promising potential in engineering.

## 1. Introduction

Cells are dynamic and complex systems in which extensive molecular networks, functioning as part of signaling pathways, regulate biological processes such as development and responses to environmental cues. Gene-specific transcription factors (TFs) are terminal components of these signaling pathways and bind to target gene promoters to induce or repress gene transcription [1]. TFs also bind other proteins such as co-activators, thereby linking genes to the general transcriptional machinery [2], often through their large intrinsically disordered transcription regulatory domains (TRDs).

Thirty to 50% of eukaryotic proteins contain large intrinsically disordered regions (IDRs) [3,4]. Despite being biologically active, intrinsically disordered proteins (IDPs) and IDRs do not adopt persistent tertiary structures by themselves. Instead, they form dynamic conformational ensembles [5,6,7,8]. Both IDRs and low complexity sequences have an over-representation of disorder-promoting residues and an under-representation of order-promoting residues [9,10]. IDRs are structurally flexible, enabling dynamic interactions with numerous partner proteins. IDPs and IDRs, therefore, often participate in protein-protein interaction networks (interactomes) involved in fast and efficient regulation of cellular responses [11,12,13]. Interactions of IDRs are often driven by short linear motifs (SLiMs), which typically consist of 3 to 11 residues [14,15,16]. The potentially small binding interface of SLiMs enable participation in transient interactions, many of which are in the low-mid micromolar *K*_d_ range [14,17]. SLiMs often coincide with molecular recognition features (MoRFs) [18,19,20]. MoRFs are structure-prone regions within longer IDRs [21], which undergo coupled folding and binding [21,22]. Together, these ID-based features enable flexible and dynamic interactions characteristic of networks operating in stress responses and other signaling events [13,23].

Plants are constantly challenged by stressors, and to cope with and adjust to these, they rely on complex transcriptional networks regulated by a large number of TFs from different TF families [24]. IDRs and IDPs are ideally suited for molecular adjustment of plants in response to changing environments, and within recent years, it has become clear that ID play important functional roles in plants (recently reviewed in [12,25,26,27,28,29]). In this review, we focus on ID in plant TFs, which are paradigmatic with respect to ID [13], by relating features and characteristics of ID to functions in plant biology.

## 2. ID in Plant Proteomes

Early global scale analysis of ID in TFs from model organisms revealed that 91% of the TFs in *Arabidopsis thaliana* contain extended (≥30 residues) IDRs, which is comparable to 92% for human TFs [30]. Relative to other proteins, eukaryotic DNA-binding proteins have an increased ID content [31]. In a recent study, the relative content of ID in 96 plant proteomes, including both monocots and eudicots, was estimated [32]. This revealed a bias among major plant clades with a higher ID content in monocots than in eudicots. In addition, the study revealed a correlation between protein role and ID content. Although IDPs are widely distributed in all domains of life, they are more frequent in eukaryotic than in prokaryotic proteomes, suggesting that ID abundance is linked to organismal complexity [4,33]. In accordance with this, studies across a number of species, including algae and land plants, indicate that the emergence of ID in TFs may correlate with evolution of plant complexity [34,35]. To conclude, ID is extensive in plant TFs, and the ID content of TFs may be linked to the capacity of establishing complex gene regulatory networks in multicellular plants.

## 3. ID in Plant TFs

### 3.1. The NAC TF Family

In this review, we focus on selected plant TF families, which have been studied with respect to ID. The families include the Nam/Ataf 1/Cuc 2 (NAC), Apetala 2/ethylene-responsive element binding factor (AP2/ERF), basic leucine zipper (bZIP), myeloblastosis (MYB), basic helix-loop-helix (bHLH), Teosinte Branched1, Cycloidea, Proliferating Cell Nuclear Factor (TCP), and auxin response transcription factor (ARF) TF families. They all follow the general domain structure of eukaryotic TFs with at least a family-designating DNA-binding domain (DBD) and a TRD [1,13].

**Figure 1:**
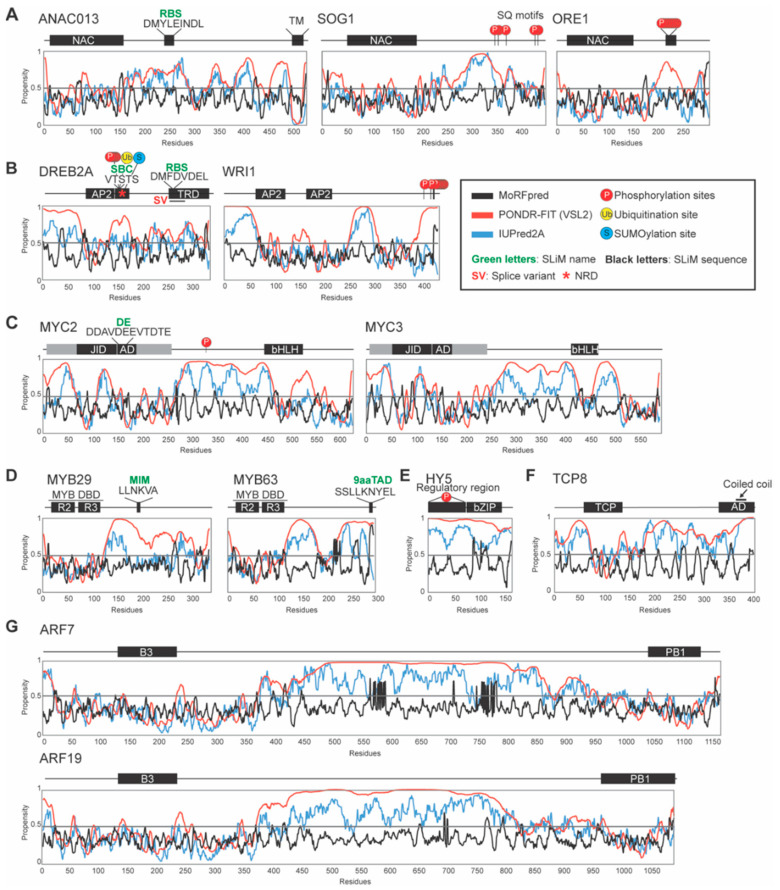
Schematic representations of the domain structures, post translational moodification sites, order-disorder profiles and MoRF profiles of plant TFs with known ID-based functions. The TF shown are from the following TF families: (**A**) NAC; (**B**) AR2/ERF; (**C**) bHLH; (**D**) MYB; (**E**) bZIP; (**F**) TCP; (**G**) ARF. ID was predicted using IUPred2A (blue line) and PONDR-FIT (VSL2) (red line), and MoRFs were predicted using MoRFpred (black line). The disorder threshold is 0.5 with disorder assigned to values larger than or equal to 0.5.

The NAC family is one of the largest plant-specific TF families with 110 genes in *Arabidopsis* [36] and 150 genes in rice [37]. NAC TFs are involved in responses to biotic and abiotic stresses and are key regulators of cross-talk between signaling pathways implicated in adaptation to environmental challenges [38,39,40,41]. In accordance with this, whole-genome expression studies in *Arabidopsis* showed that most NAC genes are induced by at least one type of abiotic stress signal [36]. Most NAC TFs consist of an N-terminal, mostly β-fold, NAC DBD [42] and long TRDs, which, a decade ago, were predicted to be intrinsically disordered [36], as shown for ANAC013 (Figure 1A) using IUPred2A [43] and PONDR-FIT [44] for disorder predictions. In addition, MoRFs were predicted using MoRFpred [45]. Since then, TRDs from *Arabidopsis* and crop NAC TFs have been shown to be mostly intrinsically disordered using biochemical and biophysical methods such as size exclusion chromatography and various types of spectroscopy [46,47,48]. Different protein conformational classes, globule, molten globule, pre-molten globule, and random coil for globular proteins, and pre-molten globule-like and coil-like for IDRs, with characteristic hydrodynamic dimensions, have been defined [13,49]. However, shape variations exist within the different classes and long IDRs may contain regions with transient secondary structure. Analysis of phylogenetically representative *Arabidopsis* NAC TRDs showed that the conformational ensembles of the ANAC046, NAP and ANAC019 TRDs correspond to pre-molten globules, whereas the ANAC013 TRD is more compact. This leaves the potential for NAC TRDs to undergo function-related changes in secondary structure and compaction. Supporting this, naturally occurring protective osmolytes, which help organisms counteract the protein denaturing effects of environmental stress, induce structure in the long ANAC046 TRD [47].

A few NAC TFs deviate from the characteristic NAC structure. Thus, Suppressor of Gamma Response 1 (SOG1) [50] belongs to a NAC sub-group of TFs containing N-terminal extensions of approximately 40 residues [36] (Figure 1A). SOG1 regulates the DNA damage response (DDR) in plants under genotoxic stress conditions [51,52], also under salinity stress. Therefore, folding and stability of SOG1 under high ionic strengths was analyzed, which showed that both the NAC DBD and the intrinsically disordered TRD play a role in the regulation of the stability of SOG1 under salinity stress [53].

### 3.2. The AP2/ERF TF Family

The AP2/ERF plant-specific TF family has 147 and 180 members in *Arabidopsis* and rice, respectively [54,55]. AP2/ERF TFs are also implicated in a variety of responses to biotic and abiotic stresses and mediate cross-talk between various signaling pathways [41,56,57]. They also hold an applicative potential in crop improvement [58]. The AP2 DBD consists of a three-stranded anti-parallel β-sheet, recognizing target genes, and an α-helix packing against one of the β-strands [59]. A recent global-scale computational analysis of AP2/ERF TFs from three model organisms, *Arabidopsis*, rice, and moss, suggested that for the *Arabidopsis* ERF1DBD, the β-strands are ordered, while the α-helical region is structurally flexible, less conserved, and of low complexity [18]. This is in accordance with the NMR solution structure suggesting that the α-helical region of ERF1 affects binding only indirectly rather than by direct promoter-binding [18,59]. The AP2 DBDs are flanked by IDRs [18], as in the case of Dehydration-Responsive Element Binding Protein 2A (DREB2A) (Figure 1B) [25], and the AP2/ERF sequences have an over-representation of disorder-promoting residues and low-complexity and homo-polymeric regions within their long IDRs [18]. The DREB2A TRD maps to a region [60], which is part of a larger DREB2A region that was experimentally shown to be intrinsically disordered and to lack secondary structure [47,61].

### 3.3. The bHLH TF Family

bHLH TFs are present in all eukaryotes and are encoded by 162 and 167 genes in *Arabidopsis* and rice, respectively [62,63]. They are key regulatory components in transcriptional networks controlling a number of biological processes, including abiotic stress responses [41,64]. The bHLH DBD consists of a basic N-terminal region involved in DNA-binding and a C-terminal dimerization region consisting mainly of hydrophobic residues forming two amphipathic α-helices [65,66]. Family members are divided into phylogenetic sub-groups based on conserved sequence motifs in their non-DBD regions [67,68]. The bHLH TF myelocytomatosis (MYC) 2 is referred to as the master of action because of its role in the regulation of cross-talk between the signaling pathways of jasmonic acid (JA), abscisic acid, salicylic acid (SA), gibberellic acid, and auxin [69]. MYC2 also contains large IDRs outside the DBD (Figure 1C). As an unusual structural feature, the N-terminal domain of MYC2, containing the activation domain (AD), is mostly structured [70] (see below).

### 3.4. The MYB TF Family

*Arabidopsis* and rice have 197 and 155 MYB TF genes, respectively [71]. The MYB TFs are characterized by sequence varying repeats R1-4 of the MYB DBDs [27,72,73], with each repeat forming three α–helices [74]. The number of MYB repeats varies, but in plants, the R2R3-type is highly enriched. The MYB TFs are key factors of development, metabolism, and responses to biotic and abiotic stresses [41,72,73]. Apart from the MYB DBD, R2R3 MYB TFs also contain large, variable regions, characterized by ID, low complexity, and numerous conserved motifs [27]. Figure 1D shows MYB29 as a representative of the R2R3 MYB TFs with the globular MYB DBD and expanded IDRs [27].

### 3.5. The bZIP TF Family

The bZIP TF family has 78 and 92 members in *Arabidopsis* and rice, respectively [75,76], and are involved in regulation of development and stress responses [41,76,77]. The family-designating bZIP domain consists of a basic region, which interacts with DNA, followed by a leucine zipper, which mediates dimerization [78]. While the basic region of bZIP TFs forms an α-helix in complex with DNA [79], this region may populate an ensemble of highly dynamic transient structures when unbound [80]. It can contain some helical structure, but lack a well-defined tertiary structure, as in the case of HY5 [81], or be completely disordered as in the case of Opaque-2 [82]. A study of the conformational ensembles of 15 different bZIP TFs suggested that the basic regions have preferences for α-helical conformations in their free forms and that intramolecular interactions between the basic regions and an eight-residue segment N-terminal of the basic regions are the primary modulators of the helicities [83]. The bZIP TFs bind DNA as dimers, and the helicities of the bZIP basic regions may affect the overall stability of the 2:1 complexes. According to a model by Hilser and Thompson [84], the degree of disorder within distinct modules may determine the nature and degree of allosteric coupling between different modules. Applying this model to the bZIP TFs, the variable intrinsic helicities within unbound monomeric bZIP basic regions may affect the leucine zipper region and influence the allosteric coupling between DNA-binding and dimerization [83].

*Arabidopsis* HY5 is a well-characterized bZIP TF acting as a positive regulator of plant photomorphogenesis. It contains a C-terminal bZIP DBD and an N-terminal regulatory region interacting with numerous protein partners, such as the E3 ubiquitin ligase COP1, to coordinate different light-modulated processes [81,85,86] (Figure 1E). HY5 is largely disordered under physiological conditions [81], and extensive biophysical analyses have shown that the N-terminal region of HY5 forms a pre-molten globular structure, while the basic region of the protein exists in a molten globule state [87].

### 3.6. The TCP TF Family

The Teosinte Branched1, Cycloidea, Proliferating Cell Nuclear Factor (TCP) plant-specific TF family contains only 24 members in *Arabidopsis*, which are mostly involved in development [88]. The TCP TFs contain a non-canonical N-terminal bHLH DBD known as the TCP domain [89]. TCP8 contains three IDRs [90], the last two of which may be fused (Figure 1F), and has hydrodynamic properties corresponding to a molten globule-like state—a relatively compact or structured state for an IDP. The N-terminal IDR overlaps with the TCP domain, particularly the basic DNA-binding region. The TCP DBD is also disordered, and most likely undergoes coupled folding and binding, when associating with the major groove of DNA [91]. A coiled coil region maps to the third region of the overall disorder, which also overlaps with the AD of TCP8, in agreement with its high content of acidic amino acid residues, characteristic of many ADs [92], and ID [90].

### 3.7. The ARF TF Family

The auxin response transcription factors (ARFs) comprise another small TF family in plants with 23 and 25 genes in *Arabidopsis* and rice, respectively [93]. ARF TFs mediate the activity of the phytohormone auxin, thereby regulating plant development [94,95]. As in the case of ARF7 and -19 (Figure 1G), most ARF TFs consists of an N-terminal B3-type DBD, a variable middle region, and a C-terminal type I/II Phox and Bem1p (PB1) domain [96]. For ARF7 and ARF19, the intrinsically disordered middle regions and the folded PB1 domains drive protein assembly formation [97], as described below.

### 3.8. Conclusions

ID is dominating in TFs from multiple TF families, as shown by both computational and experimental studies, with ID found mostly in the non-DBD regulatory regions (Figure 1). However, some free DBDs are also dynamic and intrinsically disordered and exhibit coupled folding and binding upon interactions with DNA.

## 4. MoRFs and SLiMs in Plant TF IDRs

### 4.1. SLiMs and MoRFs

IDRs evolve fast with mutational patterns differing from those of globular proteins [98,99,100,101,102]. This empowers IDRs with the plasticity needed for evolution of signaling networks. However, within IDRs, sites with secondary structure propensities are evolutionary more constrained than secondary structure regions in folded proteins [100]. Furthermore, IDRs contain multiple molecular features, e.g., increased asparagine content, that are preserved and may be associated with specific functions and can be regarded as evolutionary signatures [103]. The sites of structure propensities often coincide with SLiMs, which are present as islands of conservation within rapidly evolving regions [104], and/or MoRFs [105]. SLiMs are frequently lost and gained, also ex nihilo, at the evolutionary level [104], and both MoRFs and SLiMs are often conserved within protein sub-groups [18,19,106]. This makes both elements useful tools for determining clade-specificity. Both predicted SLiMs and MoRFs are also suggestive of functional regions within larger IDRs, typically involving interactions, making their identification extremely important. Many of the large TF IDRs are TRDs. They are therefore especially interesting with respect to predictions of interaction sites due to their interactions with co-regulators [107] and their abilities to promote phase separation of TFs [97]. While plant TF IDRs are under-studied experimentally, several of the large TF families, such as NAC, AP2/ERF and MYB, have been extensively and systematically characterized computationally [18,19,27,36].

### 4.2. Function-Related SLiM and MoRF Conservation in the NAC Family

The large and divergent intrinsically disordered NAC TRDs (Figure 1A) contain numerous SLiMs and MoRFs mapping to dips in the disorder profiles, suggestive of functional structure-prone regions [36,46,47,108]. The majority of the SLiMs are dominated by polar and negatively charged residues with conserved hydrophobic residues embedded in the polar matrix. Several of the NAC SLiMs, named WQ, W, LL, and LP (or L) (Table 1), are essential for transactivation activity [36,46,109,110]. This is also the case for the LL motif of *Hv*NAC005, which is a strong positive regulator of senescence in barley [111]. The NAC TRDs also contain numerous low-complexity histidine- and glutamine-rich regions [36].

The ID patterns of the NAC TFs are not conserved at the family level. However, specific sub-groups have conserved ID-patterns [19,36], in accordance with disorder patterns being constrained [126]. Thus, the members of the SND/VND sub-group, implicated in secondary cell wall biosynthesis [127], have conserved ID-patterns with three dips in the disorder profiles, indicative of reduced disorder, at positions coinciding with a MoRF, and the LP and WQ motifs mapping to the dip regions. For the sub-groups containing ANAC046 and ANAC013, respectively, MoRFs map to regions containing the Radical Induced Cell Death1 (RCD1)-binding SLiM (RBS) (see below) (Figure 1A,B) [108] (Table 1). The NAC TRDs also contain numerous orphan motifs representing potential functional determinants [36]. This demonstrates the use of computational analyses for the identification of functionally important regions in IDRs. Furthermore, it is apparent that many of the MoRFs hide SLiMs as functional determinants in the NAC TFs.

### 4.3. MoRFs and SLiMs Overlap in the AP2/ERF Family

Several years ago, phylogenetic analysis of the *Arabidopsis* AP2/ERF TF family revealed multiple group-specific sequence motifs in the non-DBD regions [54], and recent global-scale analyses showed that many of the numerous MoRFs conserved in these regions have unique amino acid compositions and patterns [18]. As for the NAC TFs, the most common patterns have acidic residues flanking repeated hydrophobic/aromatic residues. Some of the motifs have known functions. For example, the ERF-associated amphiphilic repression (EAR) SLiM (Table 1) functions as a transcriptional repressor in AP2/ERF and C2H2 zinc finger TFs [114,125], and the EDLL SLiM constitutes a potent acidic AD [115]. The LWSY SLiM is present in, e.g., CBF/DREB1 TFs, which play critical roles in the regulation of cold-stress related genes [128]. Thus, ectopic overexpression of *CBF1* from tomato confers increased freezing and salt tolerance and late flowering phenotype to transgenic *Arabidopsis*. The ability of the LWSY SLiM to mediate transcription depends on an aromatic SLiM-residue—tryptophan—as typical of SLiMs implicated in transcriptional activation [112]. The MCGGAI(I/L) SLiM, present in the N-termini of a group of ERF TFs [122], plays major roles in controlling flooding and hypoxia tolerance in plants and mediates regulation through N-end rule degradation of the TFs [121,129]. The RBS of DREB2A (see below) is another AP2/ERF SLiM, also present in several other TF families, with a known function (Figure 1B) [108]. All the AP2/ERF SLiMs specifically mentioned here have important biological functions (Table 1) and map to MoRF regions [18], emphasizing the importance of these IDR-related features.

### 4.4. SLiMs in the MYB Family

Conserved motifs in the non-DBD regions of MYB TFs are also useful for dividing the TFs into sub-groups with similar biological functions. In many cases, the sub-group motifs overlap with predicted MoRFs [27,73]. Several of the conserved regions overlap with functional sites. For example, the MYB sub-group 12 SLiM, (L/F)LN(K/R)(V/L)A, mediates interactions with the bHLH TFs MYC3, and MYC4 [117] (see below) (Figure 1C) (Table 1), and for MYB12, a member of sub-group 7 and a light-induced activator of flavonoid biosynthesis [130], the conserved region maps to a short C-terminal IDR [27,116].

### 4.5. Coupled Folding and Binding in the bZIP Family

So far, family-scale ID predictions for the bZIP TFs are lacking. However, the non-DBD regions also contain sequence motifs present as conserved islands in IDRs, and several of the motifs have the same characteristics as the dominating NAC and AP2/ERF motifs with conserved aromatic and/or hydrophobic residues surrounded by acidic residues [76,86]. Likely, many of the motifs overlap with MoRFs as supported by experimental analysis showing that the N-terminal domain of HY5 forms an α-helical structure in the COP1 binding region upon treatment with secondary structure inducer trifluoroethanol [81,131], and in accordance with MoRF and ID predictions (Figure 1E). The results suggest that HY5 undergoes coupled folding and binding when interacting with COP1, which targets HY5 for proteasome-mediated degradation [132]. HY5 exists in two states, phosphorylated and unphosphorylated, where the unphosphorylated state is the physiologically active state with higher affinity for target promoters [131] (Figure 1E). Phosphorylated HY5 may serve as a reserve pool that becomes unphosphorylated during the transition from dark to light for seedling photomorphogenesis [133].

### 4.6. Conclusions and Pespectives

Plant TFs contain family- and group-specific SLiMs in non-DBD regions, many of which overlap with MoRFs. Although many of the SLiMs have known biological functions, numerous remain orphans without known functions [18,27,36]. Many of these SLiMs have features typical of ADs [92] (Table 1) and may mediate interactions to regulate protein function and transcription. Supporting this interaction potential, in DELLA protein transcriptional regulators, repeated hydrophobic/aromatic residues present in conserved SLiMs of their IDRs, interact with, e.g., the GA-receptor GID1 to regulate plant development [12,134,135]. Thus, identification of interaction partners and functions remains an essential task. Recently, the understanding of ADs has improved markedly due to the increasing appreciation and understanding of ID and because of the knowledge obtained from high-throughput screenings. Generally, strong acidic ADs contain multiple clusters of hydrophobic residues near acidic residues, imposing avidity through several weak and dynamic interactions, characteristic of IDR-based fuzzy interactions [136,137]. According to a recently proposed model, acidic residues solubilize hydrophobic motifs enabling their interactions with co-activators [138]. Thus, hydrophobic motifs are balanced by acidic residues, which constitute important parts of the otherwise hydrophobic motifs. Within motifs, aromatic or leucine residues reflect the structural constraints imposed by co-activator interactions. As apparent from this review (Table 1; see below), many plant TF IDR SLiMs and SLiM regions follow these principles.

## 5. ID and Dynamics of TF-Based Interactions

### 5.1. Structural Features of the RCD1 Interactome

The structural flexible associated with ID enables proteins to participate in interactomes, which require efficient adjustments whenever needed [11,12,13]. Transcriptional networks are regulated by so-called hub proteins, which interact with several partner proteins. This hub functionality is often based on a hub with IDRs interacting with folded partners. Alternatively, the hub functionality can be mediated by a single folded domain interacting with multiple IDR-containing partners [13,107,139].

**Figure 2:**
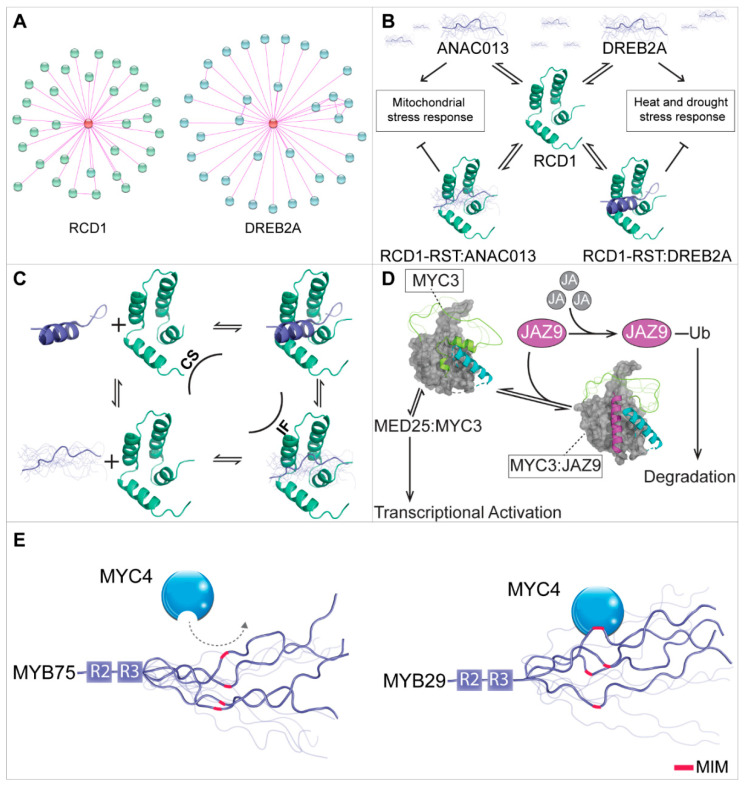
TF-ID in regulatory interactions (**A**) ID-based interactomes and mechanisms of interactions. (**A**) RCD1 and DREB2A interactomes curated from the STRING database, with medium confidence score (0.400) and including only experimental interactions. (**B**) Model of RCD1:TF interactions involving structural heterogeneity in the complexes. Several TFs, including DREB2A and ANAC013, may compete for binding to RCD1. Some TFs, e.g., DREB2A, undergo coupled folding and binding upon association with RCD1-RST, whereas other, e.g., ANAC013, may keep disorder in the complexes. RCD1 negatively regulates its TF interaction partners. The structure of the RCD1-RST domain (PDB 5OAO) is visualized using PyMOL. (**C**) Mechanisms of coupled folding and binding: conformational selection (CS) and induced fit (IF). In CS, a conformation from the TF ensemble is selected for binding by the partner protein. In IF, the TF recognizes its partner in a disordered state that folds after binding to the partner protein. (**D**) MYC3 in free (PDB 4RRU) and JAZ9-bound (PDB 4YWC) states. The Jas-motif of JAZ9 (purple) binds the N-terminal domain (grey) of MYC3, displacing the α1 helix (green) and rearranging the JID helix (cyan) of MYC3. The MYC3:JAZ9 complex cannot bind MED25 and is therefore not transcriptionally active. Upon release of JA hormone, JAZ9 is released from MYC3 and degraded through the ubiquitin-proteasome pathway enabling MED25:MYC3 complex formation. (**E**) The MYB–MIM:bHLH interaction is context dependent. The MIM SLiM, present in sub-group 12 MYB TFs as MYB29, only interacts with the bHLH TF MYC4 when present in its natural IDR context and not when inserted in the IDR context of e.g., MYB75.

The *Arabidopsis* RCD1-interactome represents a well-characterized example of an interactome with a central folded hub protein—RCD1—interacting with numerous, mostly disordered, TFs [25,140]. Some of its binding partners, e.g., DREB2A, are also hubs in large interactomes, as curated from the STRING database [141] (Figure 2A). RCD1 plays important roles in responses to reactive oxygen species, additional abiotic stressors, hormone signaling, immunity, and development [140,142,143]. The *rcd1* mutant displays pleiotropic phenotypes in development and stress responses and has a changed expression pattern of more than 400 genes [140,142]. RCD1 negatively regulates its interaction partners. Thus, downregulation of RCD1 or loss of the RBS of DREB2A, implicated in numerous abiotic stress responses [60,144], is required for proper DREB2A function under stress conditions [145]. RCD1 also negatively regulates the NAC TFs ANAC013 and ANAC017, implicated in mitochondrial stress responses. Thus, inactivation of RCD1 results in increased expression of ANAC013 and ANAC017-regulated genes from the mitochondrial dysfunction stimulon [146,147,148], further supporting a function of RCD1 in negative regulation of stress-associated TFs.

RCD1 uses the RCD1, SRO, and TAF4 (RST) domain for its many interactions with TFs [47,48,108,140].

The RST hub domain adopts a unique helical conformation consisting of four α-helices flanked by disordered segments (Figure 2B) and shares some structural features with hub domains from other proteins, such as human SIN3, CBP, and TAF4, which also play crucial roles in transcriptional regulation [149]. Thus, these small α-helical hub domains share a central structure, called αα-hairpin motif, forming a stable core of the hub domains, which accordingly were named αα-hubs. Dynamics, topologies, accessory helices, and malleability of the αα-hub domain make them unique platforms for binding intrinsically disordered TFs and ensuring signal fidelity [149].

RCD1 interacts with TFs from at least seven different TF families including the NAC, AP2/ERF, zinc finger, bHLH, bZIP, MYB, and WRKY families [108,140], all of which play central roles in signaling processes involved in development, light, and stress responses, among others [25,140]. These TFs use the RBS (DE)X(1,2)(YF)X(1,4)(DE)L [48,108] (Table 1) for binding to RCD1-RST. Although the SLiM critically depends on specific residues, including the conserved aromatic residue, for RST-binding, additional features are important for determining if a SLiM-containing region is a functional RBS. For example, the SLiM must be present in an IDR [108]. Furthermore, evolutionary conservation represents an additional filter in the identification of functional SLiMs [48].

Coupled folding and binding is paradigmatic for ID-based interactions and can take place either through conformational selection or induced fit. In conformational selection, a specific conformation from the TF ID ensemble is bound by the partner protein. In induced fit, the TF recognizes its partner in a disordered state that then folds over the surface of the partner [13,22]. Binding of DREB2A to RCD1-RST also involves coupled folding and binding (Figure 2C), although the mechanistic details remain elusive [108]. However, no α-helix induction was detected in ANAC013 and ANAC046 upon binding to RCD1-RST, despite their use of the same SLiM as DREB2A for high-affinity (low nM *K*_d_s) binding to RCD1. This suggests that the RCD1-complexes are structurally heterogeneous with the DREB2A-RBS bound as an α-helix and the ANAC013- and ANAC046-RBSs bound as disordered regions or extended structures [108] (Figure 2B). Further studies will reveal if and how the TFs, associated with similar stress responses [142], compete for RCD1 binding in regulation of stress responses despite the apparent structural heterogeneities.

The structural flexibility of the RST domain [149] likely makes RCD1 an efficient hub in the crosstalk of several pathways regulating, e.g., abiotic stress, hormonal, and immune signaling. As in the cases of TF activation domains (ADs) binding to co-regulators, negatively charged and hydrophobic amino acid residues are used by the TF-IDRs for RCD1-binding [47,108] (Table 1). Furthermore, the RST domain has characteristics, such as a positively charged patch and a hydrophobic binding pocket [149], typical of co-regulator domains binding ADs [150]. It remains to be examined, if the RCD1:TF complexes share additional features, such as dynamics in complexes [137,151], with co-regulator:TF complexes. In conclusion, the function of the large RCD1 interactome depends on a conserved SLiM-region in IDRs of TFs from several different families and formation of RCD1:TF complexes, which are structurally heterogeneous (Figure 2A–C).

### 5.2. TF IDRs in Interactions with Mediator Components: MED15:TF Interactions

The gigantic multi-subunit complex Mediator (MED) is a key transcriptional co-activator in eukaryotic cells, which links information from TFs with the central transcriptional machinery, thereby fine-tuning transcriptional reprogramming [152,153]. In plants, the MED15 subunit is important for immune responses mediated by SA [154]. MED15 is also implicated in abiotic stress responses, hormonal signaling, and development [155,156], and rice MED15 is associated with grain size [156]. MED15 contains an N-terminal folded hub domain, the KIX domain, which forms a docking site for intrinsically disordered TFs [107,139,157].

Recently, the interactome of the KIX domain of *Arabidopsis* MED15A was mapped and shown to contain 11 TFs including MYB63 (Figure 1D) and the bHLH TF UKTF1 [118]. In both MYB63 and UKTF1, the regions responsible for interactions with the KIX domain contain several copies of the Nine Amino Acid Transactivation Domain (9aaTAD), which is an important AD in eukaryotes. The motif is defined by a tandem of hydrophobic clusters, hydrophilic residues with proportional positive/negative charge, and a hydrophobic region towards its N-terminus (Table 1) [119]. Thus, in *Arabidopsis*, ADs of specific TFs target the KIX domain of MED15A for their transcriptional responses. The KIX domain, like the RST domain, is a small α-helical hub domain. In humans, intriguing mechanisms of KIX:TF interactions have been revealed. The MLL TF is capable of binding to the KIX domain simultaneously with the TF c-Myb, resulting in an allosterically enhanced affinity [158]. Binding of one TF may result in a reduced entropic cost of binding the second TF [159]. Based on this, future studies of TF binding to the KIX hub domain of MED15 are likely to reveal interesting new mechanisms of competitive and cooperative ID-based interactions.

### 5.3. Dynamics in Interactions of MYC TFs

MYC2, MYC3, and MYC4 also interact with MED as part of a complex regulatory pathway transducing JA-signals in response to a variety of biotic and abiotic stressors. Using an integrated multi-omics approach, MYC2 and MYC3 were shown to target hundreds of TFs of a gene regulatory network involving extensive cross-talk with other networks [160]. The core JA-signaling module consists of the F-box protein Coronatine Insensitive1 (COI1) [161], the jasmonate-ZIM domain (JAZ) proteins [162,163], and MYC2 [164]. COI1 is a SCF E3 ubiquitin ligase component [165], MYC2 is the master TF of JA-signaling [69,120,166], and the JAZ proteins are substrates of SCF-COI1 and transcriptional repressors of MYC2 [162,163,167,168]. In the resting state, the JA-hormone level is low, and JAZ repressors are bound to and inhibit the MYC TFs [162,163]. Upon environmental stimuli, the level of JA-hormone increases result in disassembling of the transcriptional repressor complex. The MYC TFs then recruits MED25 to mediate transcription of JA-response genes [70].

Recently, the molecular mechanism of the JAZ:MYC-regulatory unit was elucidated [70]. A conserved N-terminal region of MYC2, MYC3, and MYC4, that contains the JAZ-interacting domain (JID) [169,170] and the AD [169,171] (Figure 1C), was sufficient for interaction with JAZ9. Free, N-terminal MYC3 region forms a helix-sheet-helix sandwich fold, in which eight α-helices are positioned around a central five-stranded antiparallel β-sheet. Unusual for a free acidic AD, the MYC3 AD is structured and embedded in the helix-sheet-helix domain, forming a loop-helix-loop-helix fold with the JID and α4-helix of the AD forming a groove. While the central β-sheet is stable, the α1/α1′ helix region is very dynamic and forms only transiently, and likewise, the JID helix is dynamic (Figure 2D). Thus, two or more MYC3 conformations exist. MYC3 undergoes profound conformational changes upon binding to JAZ9. In the MYC3:JAZ9 complex, the Jas-motif forms an α-helix, which displaces the dynamic α1′/α1 helix of apo-MYC3, which in turns becomes almost completely disordered. The JID helix is also rearranged slightly in complex with JAZ. In this way, the Jas-helix of JAZ9 becomes an integral part of the structural fold (Figure 2D). Thus, protein interactions and dynamics are important for JA-perception and JAZ repression of MYC TFs.

MYC3 also directly binds to MED25 [70], thereby fine-tuning regulatory signals terminating at the promoters of MYC3 target genes [172]. Both in vitro and in planta studies suggest that the JAZ peptide competitively inhibits the interaction between MYC3 and MED25. Thus, the Jas-motif of JAZ and MED25 is likely bind to a shared MYC3 surface [70]. The Jas-motif undergoes pronounced conformational changes upon JA-hormone elicitation, and therefore switches its interactions from the MYC TFs to COI1 [70,173], freeing MYC3 for interactions with MED25. To conclude, the molecular mechanism of the JAZ:MYC:MED15 regulatory unit depends on protein dynamics, coupled folding and binding as well as coupled unfolding and binding for its function in JA-hormone signaling.

### 5.4. Synergistic MYB:bHLH TF Interactions

Synergistic or antagonistic physical interactions between plant TFs are important in the regulation of various pathways. To exemplify, synergistic interactions between R2R3-MYB TFs, bHLH TFs, and WD40 proteins form a MYB:bHLH:WD40 complex which regulates anthocyanin biosynthesis in a wide range of plant species [174]. Interactions between MYC2 and the bZIP TFs, G-box-binding factors (GBFs), result in functional antagonism in blue light-mediated *Arabidopsis* seedling development [175], and interactions between the TCP TF *Os*TB1 and the MADS TF *Os*MADS57 regulate tillering in rice [176], whereas interactions between TCP4 and WRI1 repress the transcriptional activity of WRI1 [177]. Interactions between MYB and bHLH TFs are especially common [72] and result in different phenotypic effects. The interactions between sub-group 12 MYB TFs, consisting of MYB28, MYB29, MYB76, MYB34, MYB51, and MYB122, and the bHLH TFs MYC2, MYC3, and MYC4 regulate glucosinolate biosynthesis, thereby affecting fitness upon herbivory. The MYC TFs use the same region, the JID, for interactions with the JAZ repressors [178] (Figure 1C). A short fragment of MYB29, containing the sub-group 12-defining SLiM (L/F)LN(K/R)VA, located in the center of the non-DBD region, is sufficient for interactions with MYC3 and MYC4. Combining different experimental results, Millard et al. [117] defined (L/F)LN(K/R)(V/L)A as the MYC-interaction motif (MIM) (Table 1) (Figure 1D). In planta, MYB29 without a functional MIM was unable to rescue myb29-1 mutants, and in vitro and in planta data together showed a correlation between MYC4:MYB29 interaction affinity and phenotypic output measured as glucosinolate accumulation. To examine if the MIM alone and outside of its normal protein context was sufficient for the interaction, the MIM was introduced into MYB75, which does not interact with the MYCs. However, MYB75 with an introduced MIM does not interact with MYC4 (Figure 2E). To conclude, the results suggest that not only the MIM, but also its context, such as flanking regions, the local chemical environment, the disordered structural ensemble including accessibility and compactness are important to binding [117,179].

### 5.5. IDR of TCP8 Acts as a Transactivation and Self-Assembly Domain

As described above, TCP8, which plays a role in plant-pathogen interactions [180] and flowering control [181], contains several IDRs. Of those, the C-terminal IDR contains the AD (Figure 1F). Biochemical analysis showed that TCP8 oligomerizes to form dimers, trimers, and higher-order multimers and that the C-terminal IDR is required for this self-assembly [90]. The coiled coil region inside the IDR could be responsible for oligomerization. However, multimerization of TCP8 is not only due to the coiled coil region, since speckles were also observed in biomolecular fluorescence experiments when the coiled coil region was removed from TCP8. The IRD increases aggregation of TCP8 dimers, possibly due to the high proportion of glutamines and asparagines, which are aggregation-prone residues [182]. In interactions with TCP8, TCP15 stabilizes the TCP8 monomer in an aggregation-incompetent conformation. To conclude, the C-terminal IDR of TCP8 acts both as an oligomerization domain and an AD. Moreover, many MoRFs were predicted for TCP8, indicating that this TF interacts with several protein partners to fine-tune the regulation of transcription in plant-pathogen interactions [90].

## 6. Posttranslational Modifications of TF IDRs

### 6.1. PTMs of IDRs

Posttranslational modifications (PTMs) play important roles in the regulation of cells, and IDRs are predominant sites of PTMs due to their conformational flexibility increasing the accessibility to modifying enzymes [183,184]. PTMs can regulate protein function in many different ways, which often involve changes in conformational dynamics or compaction of the IDR or the whole parent protein [184,185]. PTMs can also regulate transitions between states such as disordered and folded states [184,186] or monomeric and phase-separated states [187]. Phosphorylation, a frequent PTM, results in charge changes affecting ID-based interactions by fine-tuning both kinetics and affinities [188]. Chemically, phosphorylation replaces a neutral hydroxyl group with a phosphoryl group containing two negative charges, thereby altering the steric, chemical, and electrostatic properties of the residue and introducing new interaction potentials. Phosphorylated residues can form strong salt bridges with arginine residues, and such changes in charge may also affect the conformational ensemble of an IDR [184].

Phosphorylation and ubiquitination are two prominent PTMs controlling TF activity [189]. Increasing evidence suggests a functional overlap between primary degrons, which are peptide motifs specifying substrate recognition by E3 ubiquitin ligases [190], and ADs for unstable TFs leading to “activation by destruction”. In general, proteins with IDRs have shorter half-lives than proteins without these features [191]. An ubiquitin proteasome system (UPS)-mediated turnover of TFs is essential for their ability to activate gene transcription [189,192], and ID near a poly-ubiquitin signal serves as an initiation site for proteasomal degradation [193]. The small ubiquitin-like modifier (SUMO) is another important PTM [194]. Although the structures of ubiquitin and SUMO are similar, they may exert antagonistic functional regulation of their targets [195]. As shown below through specific examples, phosphorylation and ubiquitination of plant TF-IDRs affect various facets of the TFs including stability, cellular localization, and interactions with other proteins.

### 6.2. Phosphorylation of IDR in SOG1 Affects Target Gene Expression and DDR

**Figure 3:**
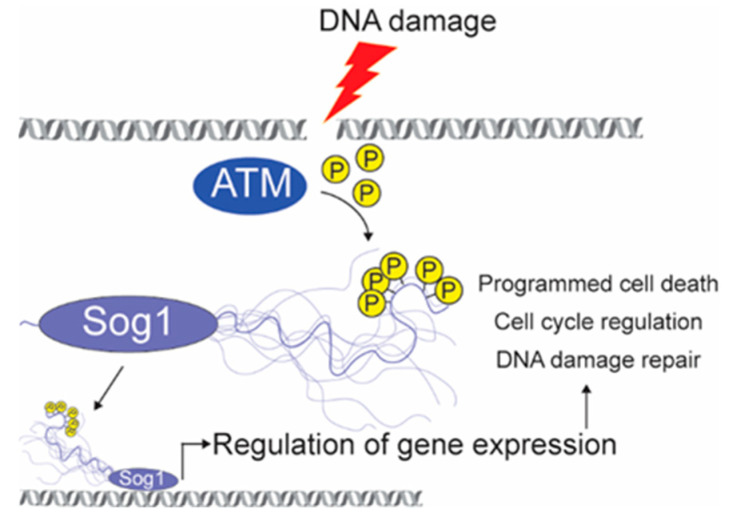
Regulation of biological function by PTMs of plant TF IDR as in the case of SOG1 phosphorylation in the DDR. Upon DNA damage, ATM (kinase) is activated and phosphorylates five SQ motifs in SOG1. SOG1 regulates the expression of genes associated with DDR, cell cycle regulation, and programmed cell death. The mechanisms of this activation of SOG1 remains unknown, but phosphorylation of the SOG1 IDR may affect the exposure of the DBD through long-range electrostatic interactions between the IDR and the DBD.

SOG1 plays essential roles in DDR, resulting in cell cycle arrest, DNA repair, and apoptosis [29,50,123,196]. SOG1 is regulated posttranslationally by Ataxia Telangiectasia Mutated (ATM) kinase in response to DNA damage by hyperphosphorylation of serine-glutamine (SQ) motifs of the C-terminal IDR of SOG1 [123,124] (Table 1). SOG1 has five SQ ATM-target motifs (Figure 1A). Initially, 350SQ and 356SQ are phosphorylated, followed by phosphorylation of the other three motifs (372, 430, 472), and the extent of phosphorylation regulates gene expression levels and determines the strength of the DDR. Thus, genes involved in DNA repair and cell cycle progression undergo gradual induction and repression, respectively, as the number of phosphorylated SQ-sites increases. Furthermore, inhibition of DNA synthesis, programmed cell death, and cell differentiation are incrementally induced as the number of phosphorylated SQ-sites increases (Figure 3) [124,196,197]. Although the molecular mechanism remains unknown, the surface of the DBD of SOG1 has been suggested to gradually become exposed with sequential phosphorylation of the SQ motifs in a manner increasing the binding affinity of SOG1 for the promoter regions of its target genes [52,124].

The functions and regulatory mechanisms of SOG1 are comparable to those of the human oncogene TF p53 [196,198]. Many of the SOG1 target genes have human orthologs that are p53 targets [52]. For example, the SOG1 target gene KRP6 encodes a cyclin-dependent kinase inhibitor resembling p21 and p27, which are important targets of p53 activity and mediate the down-regulation of cell cycle genes [51]. p53 and SOG1 are neither structurally similar nor evolutionary related and may represent functional convergence. DNA-binding by p53 is regulated by long-range electrostatic interactions between the intrinsically disordered AD and the DBD. These interactions impair binding of p53 to non-specific DNA, but not to p53-target DNA, providing a way in which p53 can discriminate between cognate and non-cognate sites in the genome [199]. Speculating, phosphorylation of the SOG1-IDR may also affect DNA-binding through changes in long-range interactions with the DBD. Furthermore, as in the case of the p53 IDRs, the IDRs of SOG1 may enable SOG1 to participate in multiple interactions with other proteins, awaiting identification, to regulate the DDR (Figure 3).

### 6.3. Calcium-Dependent Phosphorylation of ORE1-IDR Regulates the Decision to Die

Calcium-regulated protein kinases are key components of intracellular signaling in plants and mediate rapid stress-induced responses to environmental changes. Recently, an in vivo phosphoproteomics screen with inducible CPK1 identified the NAC TF ORE1 (ANAC092), a key regulator of senescence and programmed cell death [26,200], among the CPK1 phosphorylation substrates. CPK1-dependent phosphorylation of ORE1 increases transcriptional activation of the ORE1 downstream target gene BFN1, and plants overexpressing *ORE1*, undergo early senescence. A C-terminal IDR of ORE1, containing eight serine and threonine residues, was suggested to be a phosphorylation hotspot [201,202] (Figure 1A). Multiple phosphorylations introduce multiple negative charges, which could affect the ORE1 IDR in many ways, as described above. Thus, the decision to die, governed by ORE1, is regulated at the posttranslational level by phosphorylation linked to calcium-signaling.

### 6.4. Phosphorylation of AP2/ERF TFs

Disorder-based phosphorylation analyses of the AP2/ERF TFs suggest that the intrinsically disordered non-DBD regions have a higher fraction of predicted phosphorylation sites than the full-length sequences [18]. Phosphorylation of AP2/ERF TFs has also been experimentally studied, and for example the intrinsically disordered C-terminus of *Arabidopsis* ERF104 is phosphorylated by the MAP kinase MPK6, resulting in increased stability of ERF104. The ERF104:MPK6 interaction is lost in response to flagellin-derived flg22 peptide. Complex disruption requires both MPK6 activity and ethylene signaling and enables ERF104 to access target genes [203]. Several additional studies have shown regulatory phosphorylations of AP2/ERF TFs. For example, the effects of phosphorylation on functions of the AP2/ERF TFs WRINKLED1 (WRI1) and DREB2A have been studied in detail, as described below.

### 6.5. Effects of IDR in WRI1 on Protein Stability and Plant Oil Biosynthesis

WRI1 is required for the regulation of oil biosynthesis in plants [204,205], and the major part of genes with decreased expression levels in *wri1-1* encodes fatty acid and glycolytic enzymes [206]. *Arabidopsis* WRI1 contains three large IDRs [207] (Figure 1B). The C–terminal IDR of WRI1 may affect its stability, and a PEST motif is located in this IDR. PEST motifs are stretches of hydrophilic sequences that are enriched in proline, glutamic acid, serine, and threonine and function as proteolytic signals [208], and they are often associated with IDRs [209]. Phosphorylation of PEST motifs may enhance protein degradation [210], and removal of the PEST motif or mutations in putative phosphorylation sites increase the stability of WRI1 and result in increased oil biosynthesis in tobacco leaves [207]. The IDR allows exposure of the PEST motif to protein kinases and phosphorylation with structural consequences, finally affecting WRI1-mediated plant oil biosynthesis. This suggests a strategy to increase accumulation of oils in plant tissues through WRI1 engineering [211,212].

### 6.6. Phosphorylation, SUMOylation, and Ubiquitination of DREB2A IDR Regulate Heat Stress Responses

Heat stress (HS) results in enormous agricultural losses making unraveling of the HS-related networks of uttermost importance. As part of large TF networks, DREB2A is a key TF regulator of HS and drought tolerance [60,144,213,214,215]. DREB2A activity is regulated at several levels, also involving phosphorylation of the negative regulatory domain (NRD), which leads to UPS-mediated degradation (Figure 1B). Deletion of the NRD produces constitutively active DREB2A and improves heat and drought tolerance [60,144]. Phosphorylation of serines and threonines within the NRD is essential for degradation of DREB2A under non-stress conditions. The E3 ubiquitin ligase component BPM2 interacts with the NRD of DREB2A [216] through recognition of the speckle-type POZ protein-binding consensus (SBC) SLiM, ϕπS(S/T)(S/T)(S/T) (ϕ, nonpolar; π, polar) (Table 1) [113], which overlaps with the serine/threonine cluster of the NRD (Figure 1B). Molecular dynamics studies indicate that phosphorylation of a disordered segment may shift the conformational ensemble towards binding competent sub-states [217]. Under non-stress conditions, the DREB2A NRD is poly-phosphorylated resulting in degradation. During heat stress, phosphorylation, and thereby degradation of DREB2A is inhibited. Thus, the NRD is a temperature-sensitive degron with phosphorylation acting as a switch, enabling regulated ubiquitin-mediated degradation of DREB2A [218].

DREB2A is also regulated by SUMOylation [219] (Figure 1B) of Lys163, a conserved residue in the NRD [48]. This hinders DREB2A interactions with BPM2, thereby increasing DREB2A stability under high temperature. Both the phosphorylation and the SUMOylation sites map to a large IDR (Figure 1B), and in addition to the interactions with PBM2, phosphorylation of the NRD may contribute to the maintenance of a disordered structure to ensure proteasomal degradation. ID close to a poly-ubiquitin signal serves as an initiation site for proteasomal degradation by facilitating substrate unfolding and entry into the proteasome catalytic core [190]. Thus, several PTMs, mapping to IDRs, conditionally regulate DREB2A degradation as part of the regulatory mechanisms in plant HS-responses. These regulatory mechanisms are also relevant for crop plants, such as DREB2A;2, from the important legume soybean, which also has a serine/threonine-rich NRD [220].

### 6.7. Degradation-Coupled Fine-Tuning of MYC2 Activity

MYC2 is also subjected to UPS-dependent degradation, and during JA-responses the levels of MYC2 correlate positively with the expression of early wound-response genes and negatively with the expression of late pathogen-responsive genes [120]. A 12 residues acidic degron in the AD of MYC2, named DE, is required for both degradation and transcriptional activity of MYC2 (Figure 1C). Phosphorylation at Thr328 of MYC2, which is part of a large IDR and facilitates MYC2 turnover, is also important for the MYC2 function. (Figure 1C). How the two sites are functionally coupled, and how the large IDR affects this, remain elusive, but it is possible that phosphorylation recruits the UPS-machinery to destroy MYC2. In mammalians, it is generally accepted that degrons, which are usually acidic, overlap with the ADs of unstable TFs [221]. All three MYC TFs implicated in JA-signaling are regulated by E3 ubiquitin ligases, which mediate poly-ubiquitination of the TFs. The stability of the E3 ubiquitin ligase component BPM3 is enhanced by JA, establishing a negative feedback regulatory loop to control MYC levels and activity [222]. To conclude, phosphorylation and turnover of the JA-associated MYC TFs are tightly linked with their functions in regulation of the expression of JA–responsive genes.

### 6.8. Regulation by Alternative Splicing of DREB2A IDR

Early in the appreciation of ID, alternative splicing of RNA was also associated with IDRs [223]. Thus, evidence was provided that regions of pre-mRNA that are removed by alternative splicing, frequently encode regions of ID in the corresponding proteins, and that this results in functional and regulatory diversity through the various isoforms [223]. *Arabidopsis* DREB2A provides an excellent example of this. Thus, a splice variant of DREB2A, which lacks the RBS (Figure 1B), accumulates during heat stress and senescence. In addition, RCD1 is rapidly degraded during heat stress, suggesting that removal of RCD1 or the loss of the RBS of DREB2A by alternative splicing is required for proper DREB2A function under stress conditions [145].

## 7. Phase Separation

### 7.1. Membraneless Organelles

Eukaryotic organelles provide spatial control of proteins and genetic materials and can facilitate cellular reactions. In addition to membrane-enclosed organelles, eukaryotic cells also possess membraneless compartments that can be present both in the nucleus and in the cytoplasm [224]. Numerous examples of membraneless organelles have been described, including the nucleolus [225], the Cajal bodies [226] inside the nucleus, the P-bodies [227], the P granules [228] and the stress granules [229] in the cytoplasm. Membraneless organelles are usually composed of proteins and DNA or RNA, and often proteins that undergo demixing contain RNA-binding domains and low complexity sequence domains (LCDs) [230,231]. The formation of membraneless compartments or liquid-liquid phase separation (LLPS) is driven by an increase in the local protein concentrations and intermolecular interactions among the compartment body components [231,232,233]. Membraneless organelles have liquid-like properties, meaning that they are spherical in shape due to the liquid surface tension, can fuse after touching and can be deformed when subjected to mechanical manipulation [232,234,235,236]. Furthermore, phase separation can be triggered in numerous ways, including changes in ionic strength, temperature, pH, crowding agents, and RNA components [231,232,233,236].

So far, most discoveries in relation to phase separations have been made through phenotypic and cell biology observations [225,227] or because they are linked to human diseases [230,232,236]. An important question is if it is possible to predict phase separation propensity. Proteins involved in phase separation may contain specific motifs such as sequences rich in arginines and phenylalanines [233] or rich in arginines and tyrosines [237]. Phase separation can also be enhanced by the ability to form planar π-π contacts, a property that was used to develop specific predictors [238,239]. Unfortunately, it is difficult to predict phase transitions based on amino acid sequences, and it is important to emphasize that the formation of membraneless organelles is often driven by more than one protein. Consequently, the predictors developed so far are to some extent specific for a specific amino acid composition or a particular scenario, and their abilities to predict proteins involved in phase separation in proteomes are weak [239].

Previous studies provide several examples of how LLPS has been described in plants. The membraneless organelles are involved in different functions such as the regulation of RNA metabolism, translation, and protein transport, similar to what is known for animals (P-bodies and RNA granules) [240,241]. However, studies of plants have also introduced unique examples of proteins that undergo phase separation. One example is the chloroplast of *Chlamydomonas reinhardtii* that takes advantage of membraneless organelles to concentrate proteins involved in carboxylation reactions [242]. In addition, the plant membraneless organelles called photobodies contain light receptors and light signaling proteins and may have a role in post-transcriptional regulation [243]. Finally, the serine/arginine-rich protein SR45 is a splicing regulator found in *Arabidopsis* that localizes to nuclear speckles and forms liquid droplets as a function of temperature and phosphorylation [244].

TFs are usually composed by one or more DBDs and one or more ADs that are typically located within an IDR [1,13,245,246,247]. The protein-DNA binding and the presence of multivalent binding domains associated with LCDs facilitate the formation of protein-protein or protein-DNA demixing [224,248,249,250,251]. The embryonic stem cell pluripotency TF OCT4 and the yeast TF GCN4 represent recent examples of transcriptional regulation by phase separation, where the two TFs phase separate with the MED1 subunit of the MED complex, which drives transcriptional control [252].

### 7.2. Multivalent Interactions and IDRs as the Driving Forces of LLPS in Plants

**Figure 4:**
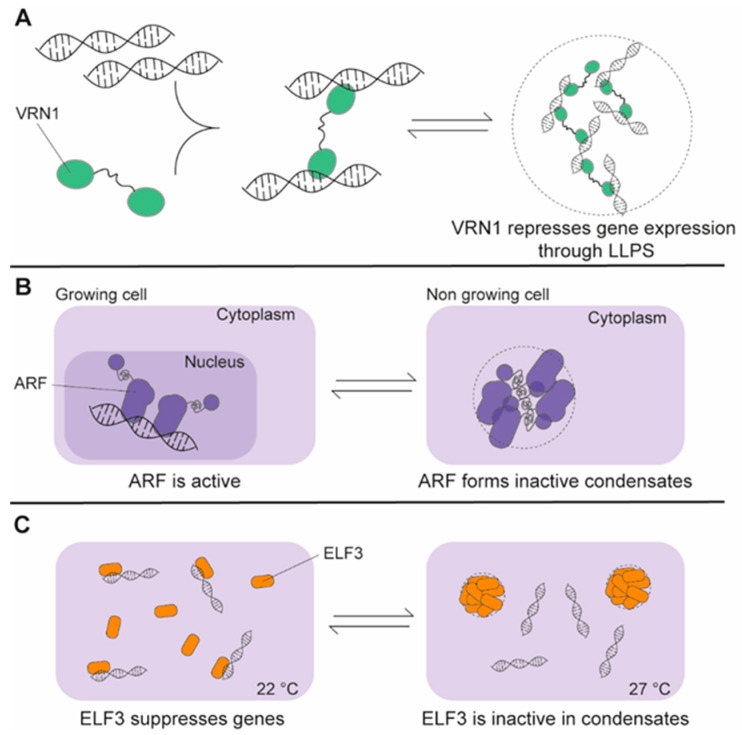
Phase separation in transcriptional regulation in plants. (**A**) The TF VRN1 undergoes phase separation in the presence of DNA to form organelles repressing gene expression. (**B**) ARF TFs promote cellular growth and differentiation through DNA-binding in the nucleus. In the stationary phase cells, the TFs are inactive in cytoplasmic condensates. (**C**) ELF3 regulation is temperature dependent: at 22 °C the protein is free and binds DNA to repress transcription, at 27 °C it undergoes protein-protein demixing to promote the genes expression.

Vernalization 1 (VRN1) is a plant TF composed by two B3-DBDs connected by a low complexity ID-region. The protein has a key role in plant vernalization [253] and undergoes LLPS with DNA [254]. In vitro DNA-binding by VRN1 is non-specific [253], and DNA and protein are both required to form the liquid droplets. This concept has already been observed for the nucleoli and RNA-binding proteins [225,240,241], for which RNA plays a regulating role. Thus, RNA serves as a scaffold or simply facilitates LLPS, thereby decreasing the local protein concentration required for demixing [255]. Both of the two B3 DBDs and the low complexity linker are essential for the phase separation [254]. This exemplifies the critical role of multivalent interactions in phase separations (Figure 4A). The example confirms that phase separation may be driven by a combination of different factors, emphasizing the complexity of this process.

### 7.3. The Cellular Localization of ARF7 and ARF19 Regulates Phase Transitions

Liquid-like properties have been considered key characteristic of membraneless organelles, whereas solid-state properties have often been related to pathological phenotype. The auxin response factor 7 and 19 (ARF7 and ARF19) (Figure 1G) form assemblies in root tissues [97]. Intriguingly, ARFs are active in the nucleus, but inactive in the cytoplasm where they form condensates (Figure 4B). The condensates show liquid- or solid-like properties depending on their age and are necessary to sequestrate the ARFs and prevent auxin responses and cell growth [97]. As for VRN1 [254], the condensates have the role of storing, and thus inactivating, the TFs. In addition, reversibility is a key property for metabolic regulation with the ability to release ARFs and restarting growth [97]. To conclude, the model described for ARF7 and ARF19 underlines the similarities with traditional organelles, such as the localization and compartmentalization, and their differences, such as reversibility.

### 7.4. Phase Separation to Cope with Environmental Changes

Recently, Jung et al. [256] showed that the *Arabidopsis* transcriptional regulator ELF3 reversibly forms liquid droplets in response to temperature increases, acting as a thermo sensor that regulates protein transcription at high temperatures. The sequence of ELF3 includes a poly-glutamine repeat that is part of a prion-like domain (PrLD), named from its sequence similarity to yeast prions [237]. PrLD is able to self-assemble and change conformation from an intrinsically disordered state to an aggregation state [257]. The EL3 poly-glutamine repeat is responsible for LLPS in response to higher temperatures [256] (Figure 4C). The function of this membraneless organelle is to capture ELF3 and promote growth and flowering. At temperatures below 22 °C, ELF3 diffuses freely and blocks transcription by binding DNA directly [256].

ELF3 LLPS does not represent the first example of membraneless organelle formation triggered by temperature changes [258]. In addition, the hypothesis of transcriptional regulation mediated by the formation of phase separated assembly is not new [259]. Examples include mammalian and yeast TFs [252] and are most likely just scratching the surface, with VRN1, ARF and ELF3 representing examples of specific mechanism for LLPS mediated transcriptional regulation in plants (Figure 4).

Studies of LLPS only began in the last decade [260], and many discoveries of phase separation in plant cells can be expected in the future. Plants have growth and transcriptional regulation strategies, which are different from those of animals and fungi, and phase transition represents an obvious way of reacting to external stress such as changes in temperature, salt, pH, and light. Improvement of predictors may help guide screening of TF libraries with respect to phase separation in transcriptional regulation.

## 8. Effects of IDRs on DNA-Binding

### 8.1. IDRs Affect DNA-Binding by TFs

The interplay between IDRs and structured domains, such as DBDs, also plays important roles in the functional regulation of TFs and can be mediated by allosteric coupling between different regions of the TFs [84,261]. Such an ID-based allosteric regulation is the result of the energetic balance within the conformational ensemble of IDRs, with, for example, binding to an IDR, resulting in ensemble redistribution signaled to different regions of the intact protein [84]. Furthermore, a recent study suggested that that promoter selection is governed by multiple specificity determinants distributed across long IDRs of TFs. IDR-associated specificity may accelerate binding-site recognition by rapidly localizing TFs to broad DNA regions surrounding these sites [262]. Thus, non-DBD IDRs are important regulators of DNA-binding by TFs.

### 8.2. Effects of IDRs on DNA-Binding by ORE1

For ORE1, it was analyzed if remote IDRs affect DNA-binding. Both the ORE1 NAC-DBD and full-length ORE1 (Figure 1A) were used in a large-scale analysis of the DNA-binding-site landscape and regulatory network of ORE1 [263]. Full-length ORE1 binds with higher affinity to an expanded range of sequences compared with the isolated NAC DBD, but the DNA-binding specificities were only slightly different. In addition to exerting allosteric regulation, modulating flexibility and spacing within the ORE1:DNA complex, the ORE1-IDR may also affect DNA-binding through long-range electrostatic interactions.

### 8.3. Long-Range Electrostatic Interactions of ANAC019-IDR Modulates DNA-Binding

**Figure 5:**
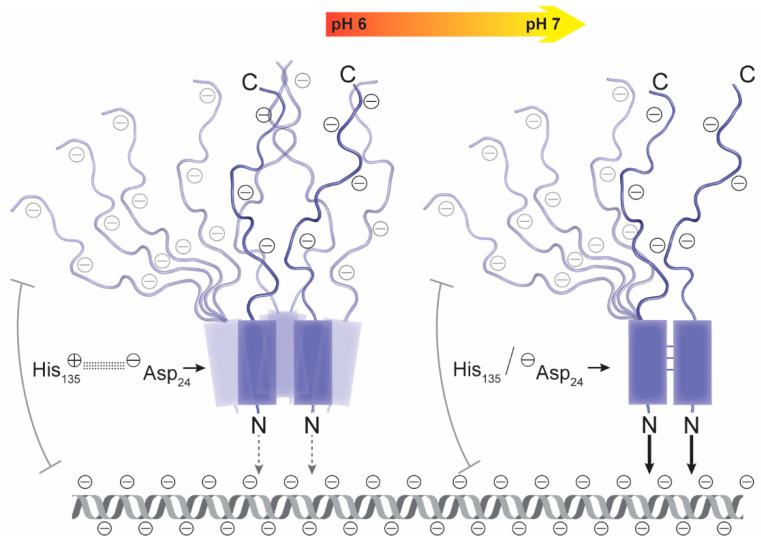
Effects of IDRs on DNA-binding. For ANAC019, a histidine switch affects DNA-binding. A “perfect” DBD (cylinder) dimer is formed, when His135 is deprotonated, which allows to stabilize interactions between residues of the two subunits of ANAC019, allowing higher affinities in DNA-binding. When His135 is protonated, a salt-bridge disrupts most of these interactions. Removal of the ANAC019 IDR abolishes the pH-dependence of DNA-binding, suggesting that the negatively charged disordered AD tunes the DNA-binding affinity of the DBD.

NAC TFs bind DNA as homo- or hetero-dimers, and dimerization is required for DNA-binding [264]. A conserved histidine switch located in the DBD of ANAC019, which is implicated in responses to both biotic and abiotic stresses [36,265], was suggested to regulate both homo-dimerization and transcriptional activity of ANAC019 [266]. The histidine switch depends on the protonation status of His135. Molecular dynamic simulations suggest the formation of a “perfect” dimer, when His135 is deprotonated, which allows for stabilizing interactions between residues of the two subunits of ANAC019 and higher affinities for target DNA. When His135 is protonated, a salt-bridge disrupts most of these interactions. Removal of the ANAC019 IDR abolishes the pH-dependence of DNA-binding, suggesting that the negatively charged disordered AD tunes the DNA-binding affinity of the DBD (Figure 5). Because of the extremely positive and negative net charge of the ANAC019 DBD and AD, respectively, a DBD only dimer would have a longer residency time near DNA than the full-length ANAC019 dimer. Thus, the DBD alone is strongly attracted to negatively charged DNA, whereas full-length ANAC019 is repelled. Long-range electrostatic interactions between DNA and the negatively charged C-terminal IDR of dimeric ANAC019 are likely to be responsible for the pH-dependence. Therefore, complex formation between the NAC-DBD only dimer and DNA is insensitive to the protonation status of His135 and, thereby, insensitive to pH changes [266].

The physiological relevance of the proposed pH-dependent DNA-binding mechanism of ANAC019 is supported by studies of the ANAC019-dependent regulation of the *ANAC083* gene, encoding a NAC TF implicated in xylem cell specification [267]. Induction of *ANAC083* expression in protoplast cells is inhibited by the His135→Lys substitution in ANAC019, which mimics protonated His135. The mutant protein is defective in dimerization and target-gene binding. This indicates that protonation of His135 in ANAC019 affects the regulation of ANAC019 target genes, also in plants. For most of the more than 100 NAC TFs [36], the net charge of the structured DBD is positive, whereas the C-terminal IDRs are negative [266]. Therefore, the pH-tuned DNA-binding mechanism of ANAC019 could be a general mechanism used by NAC TFs to orchestrate transcriptional function in responses to intracellular changes in pH [266].

## 9. Evolution of TF IDRs

### 9.1. Evolutionary Conservation of SLiM and ID Patterns in NAC TFs

Examination of the specific role of ID across different phyla can improve the functional understanding of IDRs and IDPs [29]. The history of the RCD1-interactome (Figure 2A) was traced back to the emergence of land plants with both the RCD1-RST domain and the RBS (Table 1) identified from mosses to flowering plants [48,268]. The RBS has been identified in six unrelated TF families [48,108,140] making multiple occasions of de novo evolution followed by convergence to similar functions likely [104,269]. Supporting this hypothesis, the interactions of DREB2A, ANAC013, ANAC016, and ANAC017 with RCD1 result in the down-regulation of the TF target genes [145,146] pointing to a common regulatory function of the RBS.

Alignment of orthologues and inparalogs of the 10 *Arabidopsis* TFs containing a functional RBS [108] revealed numerous additional evolutionary conserved SLiMs and sequence motifs in the non-DBD regions of the TFs, which can be regarded as homolog signatures. Disorder-order patterns are constrained compared to sequences [126], which is also the case for phylogenetically representative ANAC013, ANAC046, and DREB2 TFs [48]. The DBDs have similar ID-profiles reflecting the structured NAC or AP2 DBDs, but similar patterns are also apparent for the IDRs. The RBS-regions are associated with relatively low ID-scores in the disorder profiles, suggestive of local structure propensities. About half of the other conserved regions of the three TF groups also map to regions with low ID-scores. For the ANAC013 and ANAC046 TFs, a MoRF overlaps with the RBS-region in most of the evolutionary representative TFs. However, MoRFs are not generally conserved for the SLiM regions of these NAC and AP2/ERF TFs. To conclude, the ID-profiles of the long IDRs of the ANAC013, ANAC046, and DREB2 TFs are evolutionary conserved, and RBS maps to regions with local structure propensity.

### 9.2. MoRFs as Small Mobile Modules in AP2/ERF TF Evolution

The MoRF-oriented analysis of the AP2/ERF TF family suggests that MoRFs can be shuffled between different protein families during evolution [18]. In several cases, conserved MoRFs/SLiMs, such as LWSY, EDLL, and EAR (Table 1) occur in unrelated or distantly related families or sub-families. As part of disordered contexts, MoRFs can act as small mobile modules moving around by non-homologous recombination. When functionally advantageous, positive evolutionary selection results in preservation of the new combinations in proteins. Such mechanisms are well-suited for creation of the molecular diversities needed in complex signaling networks [18].

### 9.3. Ex Nihilo Evolution of MYB TF SLiMs

As for the NAC TFs, ex nihilo SLiM evolution is likely for the MYB family. For sub-group 12 MYB TFs, the MIM (Table 1) is responsible for the interactions with MYC3 and MYC4 [117]. The structural context of the MIM, present in an IDR, is different from that of the RB-motif, named for its interactions with R/B-like bHLH TFs [117]. This motif is located in helices 1 and 2 of the R3 repeat [270] (Figure 1D). This indicates that the MIM and the RB motifs do not have a common ancestry, and that the MYB:bHLH interactions have evolved multiple times by convergence to confer new regulatory links [117].

## 10. Conclusions

In this review, we summarize ID and protein dynamics properties for gene-specific TFs in relation to their function in plant biology. Although it is obvious that the flexibility associated with both ID and protein dynamics are advantageous for molecular functions in signaling networks of extreme biological importance, the molecular and mechanistic details remain elusive. For example, how are folded hub domains, as the RST domain of RCD1, able to bind TFs via the same TF SLiM but to different TF structures, and how does this influence putative competition scenario? To what extent are the IDRs of TFs, which are themselves hubs in the center of large interactomes, as in the case of DREB2A, responsible for the many interactions of the TF hub? For DREB2A, several molecular features of its IDRs are already known to be functionally important. Furthermore, what are the functional roles and interaction partners of the many orphan SLiMs and MoRFs of the TF IDRs? So far, many single case studies have addressed the effects of PTMs, e.g., phosphorylation, of IDRs, but consensus with respect to overall mechanistic aspects remains to be determined. For example, what are the molecular mechanisms linking phosphorylation and degradation, and do the effects of the ANAC019-AD on DNA-binding represent a generic mechanism for fine-tuning DNA-binding? Plant systems are useful models for mechanistic studies addressing these questions, since they allow analyses at the whole organismal level, as shown by effects of the SLIM-based MYB29:MYC4 interactions on glucosinolate accumulation in planta. Moreover, studies of phase separation in plants have revealed unique TF regulation pathways. Thus, plants may react to environmental changes through the formation of membraneless organelles, and additional novel mechanisms for modulations of transcription through phase separations may become apparent through additional studies. Studies of TF ID will also generally improve the basic understanding of molecular signaling in plants, and may reveal promising targets of engineering, as in the case of WRI1. Therefore, both basic and applied research in plant TF ID remains important future research goals.

## Figures and Tables

**Table 1:** Selected SLiMs and sequence motifs in IDRs of plant TFs.

Parent Protein	Family	Sequence or SLiM	Function	Name	Ref.
ANAC013	NAC	(DE)X(1,2)(YF)X(1,4)(DE)L	RCD1-interaction	RBS	[108]
CBF1/DREB1	AP2/ERF	(LV)(WYF)X(FY)	Activation	LWSY	[18,112]
DREB2A	AP2/ERF	(DE)X(1,2)(YF)X(1,4)(DE)L	RCD1-interaction	RBS	[108]
DREB2A	AP2/ERF	ϕπS(S/T)(S/T)(S/T) *	BPM2-interaction	SBC	[113]
ERF4	AP2/ERF	LDLNL	Repression	EAR	[18,114]
ERF98/TDR1	AP2/ERF	EX(4)DX(3)LX(2)L	Activation	EDLL	[18,115]
MYB12	AP2/ERF	DWX(3,10)DX(1,2)(VL)W	Activation	AD	[27,116]
MYB29	MYB	(L/F)LN(K/R)(V/L)A	MYC-interaction	MIM	[117]
MYB63	MYB	SSLLKNYEL	MED15-interaction	9aaTAD	[118,119]
MYC2	bHLH	DDAVDEEVTDTE	TA and proteolysis	DE	[120]
NAC005 (*Hv*) **	NAC	(PA)(KR)X(PC)S(LI)(ST)(ED)LL	Activation	LL	[111]
NAC013 (*Hv*) **	NAC	(FS)(IMS)(QS)LP(QP)LE(SD)P	Activation	LP (or L)	[46]
ORE1/ANAC092	NAC	L(DE)(CP)(FVI)W(NK)(YF)	Activation	W	[109]
ERF2 (*Le*) **	AP2/ERF	MCGGAI(I/L)	Degradation	***	[121,122]
SND/VND	NAC	T(DNS)W(RA)(AV)LD (KR)(FL)VASQL	Activation	WQ	[36,110]
SOG1	NAC	SQ	Phosphorylation	SQ	[123,124]
ZP1	C2H2 ZF	DLELRL	Repression	EAR	[125]

* ϕ, nonpolar; π, polar; ** *Hv*: barley, *Le*: tomato fruit. Species only shown, when not *Arabidopsis*; *** Not named.

## Abbreviations

AD	Activation domains
AP2/ERF	APETALA 2/ethylene-responsive element binding factor
ARF	Auxin response transcription factor
ATM	Ataxia-Telangiectasia-Mutated
bHLH	Basic helix-loop-helix
bZIP	Basic leucine zipper
DREB2A	Dehydration-Responsive Element Binding Protein 2A
DBD	DNA-binding domain
DDR	DNA damage response
EAR	ERF-associated amphiphilic repression
IAA	Auxin
IDR	Intrinsically disordered regions
ID	Intrinsic disorder
JA	Jasmonic acid
JID	JAZ-interacting domain
MED	Mediator
MoRF	Molecular recognition features
MYB	Myeloblastosis
MYC	Myelocytomatosis
NAC	NAM/ATAF1/CUC2
PB1	Phox and Bem1p
PrLD	Prion-like domain
PCF	Proliferating Cell Nuclear Factor
PTM	Posttranslational modification
RCD1	Radical Induced Cell Death1
RBS	RCD1-binding SLiM
SA	Salicylic acid
SQ	Serine-glutamine
SLiM	Short linear motif
SUMO	Small ubiquitin-like modifier
SOG1	Suppressor of Gamma Response 1
TA	Transcriptional activation
TCP	Teosinte branched1, cycloidea, proliferating cell nuclear factor
TF	Transcription factors
TR	Transcriptional repression
TRD	Transcription regulatory domain
UPS	Ubiquitin proteasome system
VRN1	Vernalization 1
